# Nurses’ knowledge of, attitudes towards and awareness of the metaverse, and their future time perspectives: a cross-sectional study

**DOI:** 10.1186/s12912-024-02048-y

**Published:** 2024-06-20

**Authors:** Eda Ergin, Turgay Yalcinkaya, Sebnem Cinar Yucel

**Affiliations:** 1 Faculty of Health Sciences, Department of Fundamentals of Nursing, İzmir Bakırçay University, İzmir, Türkiye Türkiye; 2 Faculty of Health Sciences, Department of Nursing, Sinop University, Sinop, Türkiye; 3 Faculty of Nursing, Department of Fundamentals of Nursing, Ege University, İzmir, Türkiye

## Abstract

**Background:**

The metaverse is a new and developing technology used in the field of healthcare. The perception of future explains time as a psychological phenomenon rather than a physical one. This study aimed to determine nurses’ thoughts of the metaverse and their perceptions of future.

**Methods:**

The study in which the cross-sectional descriptive design was used was conducted with nurses working in a hospital in Trkiye from September 2022 to December 2022. Face-to-face interviews were conducted with 374 nurses who were chosen using the convenience sampling method. Personal Identification Form, Metaverse Scale (MS) and Future Time Perspective Scale (FTPS) were used to collect data. The Statistical Package for Social Sciences (SPSS) for Windows 25.0 program was used to analyse the data.

**Results:**

The findings revealed that 81.6% of the nurses believed that they could provide patient education using the metaverse in the future, whereas 46% believed that they could do virtual nursing. The mean scores obtained from the FTPS and MS by the nurses were 3.45 (*SD* = 0.37) and 3.74 (*SD* = 0.56), respectively. There was a weak positive relationship between perception of future, and knowledge of, attitudes towards and awareness of the metaverse (*r* = 0.157, *p* = 0.002), and a weak, positive relationship between internet use duration and MS (*r* = 0.169, *p* = 0.001).

**Conclusions:**

This study underscores the potential of the metaverse in nursing, revealing that nurses are optimistic about its application in patient education and virtual care. We recommend the development of specialized training programs to equip nurses with the necessary skills and knowledge to effectively utilize the metaverse in healthcare settings.

**Supplementary Information:**

The online version contains supplementary material available at 10.1186/s12912-024-02048-y.

## Introduction

The term ‘metaverse’ first appeared in Stephenson’s science fiction novel ‘Snow Crash’, which was released in 1992. In the novel, the metaverse is a parallel universe made up of computer graphics that users can access via glasses and headphones. Users interact with the metaverse via avatars that are linked to customizable digital bodies [1]. The metaverse is defined as a decentralized, three-dimensional virtual reality environment that serves as the internet’s next generation [2]. However, there is a lack of agreement between authors in the literature regarding the definition of the metaverse, but in recent publications, it has been reported that the metaverse differs from augmented reality and virtual reality (VR) [3].

The future time perspective (FTP) is concerned with the perception of time rather than the actual physical time recorded by a calendar or clock. The time frame that individuals consider when making decisions is defined by the temporal context (i.e. how much people plan for the future), the clarity of people’s perceptions of their future needs and the extent to which the present time is connected to the past and future [4]. A strong future time perspective can include a focus on long-term goals, professional development plans and a desire for continuous growth and learning.

Intentions influence attitudes towards behavior, and intentions cause differences in behavior, according to the theory of planned behavior [5]. According to Zimbardo and Boyd [6], the time perspective (TP) theory has a widespread and powerful influence on many human behaviors. However, the time factor influences people’s interpretation of various actions to create continuity [7]. With the advent of a new technology, such as the metaverse, in the field of nursing, there may be a relationship between nurses’ knowledge of, attitudes towards and awareness of metaverse regarding their future use of this technology; thus, we conducted the present study.

## Background

The emergence of the concept of metaverse is expected to increase research and implementation of VR technology in nursing, as well as the connections between different disciplines in this field [8]. For example, patients can access 12-lead electrocardiograms, blood pressure and pulse monitors and pulse oximetry results by integrating the metaverse with existing telehealth services [9]. Virtual care models developed for chronic conditions such as hypertension, adult diabetes, morbid obesity whose management is ineffective and costly with traditional care delivery models may offer advanced interactive options in a future medical metaverse [10].

There are few studies on the use of FTP in nursing. Several studies conducted with nursing students have reported a relationship between students’ future time perspective and their career maturity [11] and professional values has been reported [12]. However, in the literature, there are some other studies in which a link was shown between FTP and technological behaviors. Miceli et al. [13] found that students’ TP was a strong predictor of Facebook addiction in a study conducted with undergraduate students. Excessive use of social media was determined to be related to future time perspective in a study of adolescents [14]. According to Przepiorka and Blachnio [14], future orientation is a negative predictor of internet and Facebook addiction. According to Lukavska [15], more game time was associated with a lower level of future time perspective.

In recent years, there has been an increasing interest in performing various healthcare services by using the metaverse [16], which is expected to play a significant role in the digital transformation of healthcare [17]. Nurses are expected to use metaverse and other future technological developments (e.g., artificial intelligence and robotics) in their nursing practices and to determine at what stage these technologies will be effective in their applications in the field of healthcare [18]. For nurses working in hospital settings, the metaverse can be viewed as an innovative environment that offers opportunities for improved patient care, professional development, and interdisciplinary collaboration. However, as far as we know, in the literature, there is no research in which nurses’ knowledge of, attitudes towards, and awareness of the metaverse and their future time perceptions are investigated. Understanding nurses’ future time perspectives and predicting their adaptation to rapidly evolving health technologies may influence their willingness to adopt metaverse technologies and thus the quality of patient care and innovation processes in healthcare institutions.

The present study was aimed determining the relationship between nurses’ future time perspectives and their knowledge of, attitudes towards and awareness of the metaverse, as well as the factors influencing this relationship. The research questions are as follows:

### Research question 1

Are nurses’ future time perspectives and Metaverse Scale scores influenced by their demographic characteristics?

### Research question 2

Is there a link between nurses’ perspective of the future and their knowledge of, attitudes towards and awareness of the metaverse?

## Methods

This descriptive and cross-sectional study was reported using the STROBE checklist [19].

## Participants

The study population included 750 nurses working in the surgical and internal medicine departments of a medical faculty hospital in western Türkiye. In the study, the convenience sampling method was used. We calculated the minimum sample size as 255 using the OpenEpi Info Statcalc™ (Version: 3.01) software (confidence interval: 95%, margin of error: 5%). However, considering the possibility of refusals (withdrawals) and/or losses during the study, we decided to include more nurses in the study (*N* = 391). Of these nurses, 17 were excluded from the study because they filled in the questionnaires incompletely. Thus, in the sample of the study, 374 nurses who met the inclusion criteria were included.

Inclusion criteria: (a) volunteering to participate in the study, (b) holding a nursing title and (c) being present in the hospital during data collection Of the nurses, those working in outpatient clinics, administrative units and operating rooms, and those who did not answer questions or complete the survey due to health issues were not included in the study.

## Data collection

After obtaining ethical approval (decision date: June 22, 2022, decision number: 639/619), the researcher collected data for the study using the face-to-face and pencil-and-paper techniques from September 2022 to December 2022. The researcher visited the clinics at regular intervals. Before taking part in the study, the nurses were reminded that their participation was voluntary and that the data would be kept confidential. The nurses were given the survey forms after the purpose of the study was explained to them and their informed consent was obtained. It took the participants to fill in the tools approximately 15–20 min.

## Data collection tools

Personal Information Form, Metaverse Scale (MS) and Future Time Perspective Scale (FTPS) were used to collect the study data.

### Personal Information form

Personal Information Form (Supplementary Material I), developed by the researchers, was used to gather the sociodemographic data of the nurses. This form consists of 13 questions. The form’s content validity index (CVI) was assessed by five nursing faculty members who were specialized in nursing education. The scope validity was assessed using a four-point Likert-type scale. Responses’ rating ranged from 4 (very relevant) to 1 (not relevant) for each question. The CVI was calculated as 0.91. A value of 0.90 or higher indicates good content validity [20].

### Metaverse scale

The goal in this study was to use the MS to determine nurses’ knowledge of, attitudes towards and awareness of the metaverse. Süleymanoğulları et al. [21] performed the validity and reliability study of the MS. The scale has 15 items and the following four sub-dimensions: technology (items 1-2-3-4-5-10-13), digitalization (items 9-11-12), social (items 14–15) and lifestyle (6-7-8). The Cronbach’s *α* value of the scale was 0.813 in Süleymanoğulları et al.’s study [21], and 0.723 in the present study, which indicates that it is an acceptable and valid tool [22]. Responses given to the items in the scale are rated on a five-point Likert-type scale, ranging from 1 = d*efinitely disagree* to 5 = *definitely agree*. The mean score of a sub-scale is calculated by dividing the scores given to all the items by the number of the items. The higher the score obtained from a subscale is the higher the level of what is measured by that subscale. The level of knowledge, attitude and awareness of participants related to the metaverse increases as the scale scores increase [21].

### Future time perspective scale (FTPS)

The FTPS was used to assess nurses’ future time perspective. Husman and Shell [23] developed the scale. Avcı and Erden (2009) conducted the validity and reliability study of the Turkish version of the FTPS. The Cronbach’s *α* value of the FTPS was 0.78 in Avcı and Erden’s [24] study and 0.898 in the present study, which indicates that it is an acceptable and valid scale [22]. There are 27 items and the following four sub-dimensions in the FTPS: Connectedness (items 1, 5, 7, 8, 12, 13, 17, 19, 21, 23, 25 and 27), value (2, 6, 9, 14, 15, 18 and 22), speed (11, 16 and 26) and extension (3, 4, 10, 20 and 24). Of the items in the FTPS, 12 (items 1, 7, 8, 11, 13, 15, 16, 17, 23, 25, 26 and 27) are reverse scored. Responses given to the items in the FTPS are rated on a five-point Likert type scale ranging from 1 (*definitely disagree)* to 5 (*definitely agree)*. The mean score of a sub-scale is calculated by dividing the scores given to all the items by the number of the items. The higher the score obtained from a subscale is the higher the level of what is measured by that subscale [23, 24].

## Data analysis

The data collected in the study were analysed using the Statistical Package for Social Sciences (SPSS) for Windows 25.0 software. Descriptive statistical methods were used to analyse the data (number, percentage, minimum–maximum values, arithmetic mean and standard deviation). Whether the data were normally distributed was checked using the Q–Q plot [25]. The skewness and kurtosis values for normal distribution should be within ± 3 [26].

An independent *t* test was used to compare numerical data in normally distributed data when comparing two independent groups, and one-way analysis of variance was used when comparing more than two independent groups. To test the relationship between numerical variables, Pearson correlation was used. The correlation coefficient was calculated as follows: 0.00–0.10 (negligible correlation), 0.10–0.39 (weak correlation), 0.40–0.69 (moderate correlation), 0.70–0.89 (strong correlation) and 0.90–1.00 (very strong correlation) [27].

## Results

### Demographic characteristics

The mean age of the nurses was 29.54 ± 6.53 years, with a mean length of service of 7.09 ± 6.70 years. Their average daily internet use was 4.98 ± 3.60 hours.

Of the nurses in the study, 82.6% were female, 54.3% held a bachelor’s degree, and 38% held a graduate degree. The nurses use Instagram most (92.5%) and Facebook least (38.8%). Among the nurses who participated in the study, 77.8% had heard the term ‘metaverse’ before, 71.4% understood what it meant, 81.6% believed that patient education could be given in metaverse in the future, and 46% believed that virtual nursing could be performed through metaverse in the future (Table 1).

**Table 1:** Comparison of FTPS and Metaverse Scale scores’ mean values according to the sociodemographic characteristics of nurses (*N* = 374)

Variables	*n* (%)	MS		FTPS	
		Mean ± SD	Test and *p*	Mean ± SD	Test and *p*
Gender					
Female	309 (82.6)	26.51 ± 3.71	*t* = − 0.772 *p* = 0.440	3.45 ± 0.38	*t* = 0.339 *p* = 0.735
Male	65 (17.4)	3.73 ± 0.8		3.44 ± 0.39	
Educational level					
High school	29 (7.8)	3.61 ± 0.78	*t* = 1.927 *p* = 0.382	3.46 ± 0.34	*F* = 1.002 *p* = 0.368
Bachelor’s degree	203 (54.3)	3.74 ± 0.57		3.43 ± 0.37	
Master’s degree	142 (38.0)	3.78 ± 0.51		3.48 ± 0.4	
Usage of Facebook					
Yes	145 (38.8)	3.73 ± 0.61	*t* = − 0.113 *p* = 0.910	3.43 ± 0.34	*t* = − 0.988 *p* = 0.324
No	229 (61.2)	3.75 ± 0.54		3.47 ± 0.4	
Usage of Instagram					
Yes	346 (92.5)	3.74 ± 0.57	*t* = − 0.322 *p* = 0.747	3.44 ± 0.37	*t* = − 1.781 *p* = 0.076
No	28 (7.5)	3.78 ± 0.46		3.57 ± 0.41	
Usage of Twitter					
Yes	218 (58.3)	3.77 ± 0.58	*t* = − 1.511 *p* = 0.131	3.49 ± 0.38	*t* = 2.424
No	156 (41.7)	3.71 ± 0.54		3.39 ± 0 0.38	***p*** = **0.016**
Usage of LinkedIn					
Yes	156 (41.7)	3.82 ± 0.46	*t* = − 2.092 ***p*** **= 0.036**	3.55 ± 0.37	*t* = 4.441 ***p*** = **0.000**
No	218 (58.3)	3.69 ± 0.63		3.38 ± 0.37	
Have you heard the term ‘metaverse’ before?					
Yes	291 (77.8)	3.80 ± 0.52	*t* = − 3.598 ***p*** **= 0.000**	3.47 ± 0.35	*t* = 1.957 *p* = 0.051
No	83 (22.2)	3.55 ± 0.68		3.38 ± 0.45	
Do you know what the metaverse is?					
Yes	267 (71.4)	3.82 ± 0.52	*t* = − 4.111 ***p*** **= 0.000**	3.47 ± 0.35	*t* = 1.728 *p* = 0.085
No	107 (28.6)	3.56 ± 0.64		3.4 ± 0.44	
In the future, could patient education be provided through the Metaverse?					
Yes	305 (81.6)	3.82 ± 0.52	*t* = − 5.397 ***p*** **= 0.000**	3.48 ± 0.38	*t* = 3.545 ***p*** **= 0.000**
No	69 (18.4)	3.4 ± 0.63		3.31 ± 0.34	
In the future, is it possible to perform virtual nursing using Metaverse?					
Yes	172 (46.0)	3.85 ± 0.6	*t* = − 3.817 ***p*** **= 0.000**	3.48 ± 0.40	*t* = 1.630 *p* = 0.104
No	202 (54.0)	3.65 ± 0.52		3.42 ± 0.36	

Note FTPS, Future Time Perspective Scale; MS, Metaverse Scale; *SD*, standard deviation. Statistical significance is highlighted in bold

### Mean scores obtained from the overall FTPS and MS, and their sub-dimensions

The mean score obtained from the FTPS by the nurses was 3.45 ± 0.37. The nurses obtained the highest mean score from the value sub-dimension (3.91 ± 0.64) and the lowest mean score from the speed sub-dimension (2.94 ± 0.67). The mean score obtained from the MS by the nurses was 3.74 ± 0.56. The nurses obtained the highest mean score from the lifestyle sub-dimension (3.96 ± 0.70) and the lowest mean score from the digitalization sub-dimension (3.49 ± 0.81) (Table 2).

**Table 2:** Average scores of FTPS and MS total and subdimension scales (*N* = 374)

Scales and subdimensions	Min	Max	$\bar X \pm SD$
FTPS total score	1.00	5.00	3.45 ± 0.37
Connectedness	1.00	5.00	3.91 ± 0.64
Value	1.00	5.00	2.94 ± 0.67
Speed	1.00	5.00	3.44 ± 0.96
Extension	1.00	5.00	3.04 ± 0.61
MS total score	1.00	5.00	3.74 ± 0.56
Technology	1.00	5.00	3.77 ± 0.58
Digitalisation	1.00	5.00	3.49 ± 0.81
Social	1.00	5.00	3.69 ± 0.93
Lifestyle	1.00	5.00	3.96 ± 0.70

Note$\bar X$, mean; *SD*, standard deviation

### Comparison of the mean scores obtained from the overall FTPS and metaverse scale according to sociodemographic characteristics of nurses

In our study, of the nurses, those who used Twitter obtained higher scores from the FTPS and those who used LinkedIn obtained higher scores from the MS and FTPS (*p* < 0.05, Table 1).

In the present study, it was determined that nurses who had previously heard of the metaverse and knew what it was and who believed that patient education and virtual nursing could be performed in the metaverse obtained higher scores from the MS. Furthermore, of the nurses, those who believed that virtual nursing could be performed in the future obtained higher scores from the FTPS (*p* < 0.05, Table 1).

### Correlation analysis

There was a weak negative correlation between the scores the nurses’ obtained from the value sub-dimension and the variables such as age and professional experience, (*r* = − 0.167, *p* < 0.01; *r* = − 0.165, *p* < 0.01, respectively). There was a weak positive correlation between the daily internet use variable and the scores the nurses’ obtained from the value sub-dimension (*r* = 0.104, *p* < 0.05), the extension sub-dimension scores (*r* = 0.101, *p* < 0.05), the Metaverse Scale (*r* = 0.169, *p* < 0.01), the technology sub-dimension (*r* = 0.151, *p* < 0.01), the digitalization sub-dimension (*r* = 0.166, *p* < 0.01) and the lifestyle sub-dimension (*r* = 0.114, *p* < 0.05; Table 3).

**Table 3:** Correlation between the scales and subdimensions with continuous variables (*N* = 374)

Scales and subdimensions	Age	Years of professional experience	Daily internet usage time
	*r*		
FTPS total score	−0.089	−0.088	−0.005
Connectedness	−0.034	−0.044	−0.033
Value	−0.167^**^	−0.165^**^	0.104^*^
Speed	0.073	0.091	−0.062
Extension	−0.019	−0.015	0.101^*^
MS total score	−0.074	−0.087	0.169^**^
Technology	−0.082	−0.094	0.151^**^
Digitalisation	−0.085	−0.093	0.166^**^
Social	0.026	0.019	0.076
Lifestyle	−0.060	−0.074	0.114^*^

Note Pearson correlation coefficient (*r*)

^*^p < 0.05

^**^p < 0.01

A positive, weak significant correlation was determined between the scores the nurses obtained from the FTPS and MS (*r* = 0.157, *p* < 0.01). There was a positive, weak significant correlation between the scores the nurses obtained from the overall FTPS and the lifestyle (*r* = 0.180), technology (*r* = 0.143) and digitalization (*r* = 0.110, *p* < 0.05) sub-dimensions of the MS. There was also a positive, weak significant correlation between the scores the nurses obtained from the overall MS and the extension (*r* = 0.183) and value (*r* = 0.181) sub-dimensions of the FTPS, and a negative, weak significant correlation between the scores the nurses obtained from the overall MS and the speed sub-dimension of the FTPS (*r* = -0.104, *p* < 0.05) (Table 4).

**Table 4:** Results of correlation analysis between scales and subdimensions (*N* = 374)

Scales and subdimensions	1	2	3	4	5	6	7	8	9	10
	*R*									
1. FTPS total score	1									
2. Connectedness	0.794^**^	1								
3. Value	0.507^**^	−0.027	1							
4. Speed	0.470^**^	0.531^**^	0.177^**^	1						
5. Extension	0.117^*^	0.316^**^	0.378^**^	0.433^**^	1					
6. MS total score	0.157^**^	0.063	0.181^**^	−0.104^*^	0.183^**^	1				
7. Technology	0.143^**^	0.074	0.148^**^	−0.054	0.113^*^	0.932^**^	1			
8. Digitalisation	0.110^*^	−0.010	0.231^**^	0.165^**^	0.188^**^	0.789^**^	0.673^**^	1		
9. Social	0.049	−0.015	0.044	−0.052	0.179^**^	0.480^**^	0.281^**^	0.148^**^	1	
10. Lifestyle	0.180^**^	0.131^*^	0.129^*^	−0.072	0.139^**^	0.852^**^	0.757^**^	0.562^**^	0.321^**^	1

^*^p < 0.05

^**^p < 0.01

## Discussion

### Demographic characteristics

In the present study, the aim was to determine nurses’ future time perspective and metaverse-related views.

In this study, we aimed to determine nurses’ future time perspectives along with their knowledge, attitudes, and awareness of the metaverse. Although the metaverse is a relatively new concept in the healthcare sector [28], the findings of this study show that the majority of nurses (77.8%) have heard of it and know what it is (71.4%). According to statistical research, 31% of adults in the United States have heard of the metaverse but do not know much about it [29], and 52% of individuals in 29 different countries have knowledge of the metaverse [30]. This discrepancy underscores the potential readiness of healthcare workers, particularly nurses, to engage with new technologies and integrate them into their practice. The increased awareness of metaverse among nurses, compared to the general population can be attributed to the proactive engagement of health sectors with innovative technologies and their impact on future medical and nursing education and practice.

In the present study, it was determined that nearly half of the nurses responded positively to the question “Can virtual nursing be performed using the metaverse?” A study conducted with nursing students who would be the nurses in the future revealed that the metaverse could help their clinical nursing practices [31]. There are studies in the literature that show virtual nursing benefits nurses [32–34]. Furthermore, more than half of the nurses in our study said yes to the question “Can the metaverse can be used for patient education?” Our review of the literature demonstrated that the metaverse could be used in nursing education [35], monitoring of patients’ health status at home [36], lung cancer surgery training for doctors [37] and education of various university students [38]. These findings highlight the potential of the metaverse to revolutionize nursing practice and encourage innovative approaches to patient care and education in the digital age.

### Factors influencing the knowledge, attitude, awareness and future-oriented perspectives of nurses in the metaverse

In 2021, Facebook changed the word metaverse to ‘Meta’ and had a large impact [39], but in our study, we discovered that the mean MS scores of the nurses who used the LinkedIn social network were higher. This result can be explained by the fact that health-related information is shared more frequently on LinkedIn than on other social networks. Furthermore, it was discovered that nurses who had previously heard of or known about the metaverse obtained higher mean scores from the MS. In studies in which the metaverse knowledge levels of physical education and sports teacher candidates were investigated, it was determined that of the students, those who knew the term ‘metaverse’ obtained higher scores from the Metaverse Scale [40]. Therefore, by organizing metaverse training for nurses on what the metaverse is and how to use it before they begin using the prepared metaverse platforms, one can increase nurses’ knowledge of, attitudes towards and awareness of the metaverse.

The metaverse should be viewed as an environment for long-term education, free of time and space constraints, with the potential to turn imagination into reality through the convergence of artificial intelligence-supported advanced technologies [3]. In this configuration, it is also believed that patient education can be transferred to a virtual environment. Our findings also revealed that the MS scores obtained by the nurses who believed that patient education and virtual nursing could be performed in the metaverse were an expected outcome.

The FTPS scores obtained by the nurses using Twitter and LinkedIn were higher. Furthermore, those who believed that patient education could be provided in the future using the metaverse obtained higher scores from the FTPS.

### Correlation analyses

The value concept of the FTPS can be defined as making efforts for future goals now [4]. According to Weikamp and Göritz [41], as the perceived duration increased in terms of age and profession, so did the perspective of the future in a professional context. According to the literature, the value sub-dimension of the FTPS was negatively weakly related to nurses’ age and professional experience duration. Furthermore, the value sub-dimension of the FTPS had a negative, weak relationship with the MS, suggesting that pilot applications be carried out on younger and less experienced nurses in metaverse platforms that are under consideration for improvement. In their study in which university students’ metaverse-oriented thoughts were investigated, Özdemir et al. [42], reported that there was a weak, positive relationship between average daily social media usage time and technology sub-dimension scores. This finding indicates that as nurses’ internet usage increases, so will their knowledge, attitude and awareness of the metaverse.

The study’s findings revealed a weak, positive correlation between the FTPS and MS, implying. that as nurses’ perspectives of future time improve, they may become more open to the idea of using metaverse in nursing care, viewing it as a valuable technology for improving future patient care. The strongest correlation was found between the extension sub-dimension of the FTPS and MS, which is defined as the perspective of existing goals being more visible and important to the individual [4]. Nurses with higher extension scores are more likely to be involved in the metaverse. Finally, in the present study, the strongest link was determined between the ‘lifestyle’ dimension of the MS and FTPS. This finding may imply that as nurses’ perspectives of future time improve, so will their lifestyle scores, which include activities, such as participating in metaverse-based activities, positively impacting their health and virtual communication.

### Implication for nurses, researchers and policymakers

In light of our findings, we recommend several avenues for future research and implications for practice. First, nursing researchers and administrators may consider developing and implementing comprehensive metaverse educational programs. Training may focus not only on the technical skills required to navigate metaverse platforms, but also on ethical considerations related to virtual patient care, data security, and patient privacy. Second, researchers can perform pilot metaverse initiatives using metaverse platforms for remote patient education or virtual nursing procedures. To achieve this goal, researchers may consider using different metaverse programs such as Meta Horizon, Microsoft Mesh, Spatial. Third, in future research, specific barriers and facilitators to the adoption of metaverse technologies among nurses could be investigated. Fourth, through a multidisciplinary approach, researchers, along with technology developers and healthcare administrators, can collaborate with nurses to consider research findings and design metaverse platforms that are user-friendly and meet the specific needs of nursing practices.

### Limitations

There are several limitations to this study. First, it was conducted in a single hospital in Türkiye, so its results are applicable only to those surveyed and they cannot be generalized to nurses working in other regions of Türkiye and other countries. Second, the study’s cross-sectional design allows for the examination of relationships between variables but not of cause-and-effect relationships. More complex longitudinal designs may be used in future research.

## Conclusion

According to our findings, there is a weak positive relationship between nurses’ future TPs and their attitudes towards the metaverse. In general, the positive correlation between future TP and attitudes towards new technologies, such as the metaverse, suggests that a person’s future-related views shape their attitude and beliefs towards such technologies. More than half of the nurses had heard of the metaverse or they were familiar with it, and they believed that it could be used for future patient education and virtual nursing care. As a result, we believe that metaverse platforms could be used in clinical settings in the future for issues, such as nursing care and patient education.

### Electronic supplementary material

Below is the link to the electronic supplementary material.

Supplementary Material 1

## Data Availability

The datasets used and/or analysed during the current study are available from the corresponding author on reasonable request.
