# Supplementary material for: Nurses’ knowledge of, attitudes towards and awareness of the metaverse, and their future time perspectives: a cross-sectional study
Source: BMC Nurs. 2024 Jun 20;23:414. doi: 10.1186/s12912-024-02048-y (PMC11188271; doi:10.1186/s12912-024-02048-y)
Supplement: Supplementary file 1 — Supplementary Material 1 [file 12912_2024_2048_MOESM1_ESM.docx]

**Supplementary Material I: Personal Information Form**

1. Gender:
2. Age:
3. Level of education:
4. How many years have you been practicing nursing professionally?
5. How many hours per day do you use the internet?
6. Do you use Facebook?
7. Do you use Instagram?
8. Do you use Twitter?
9. Do you use LinkedIn?
10. Have you heard of the term 'metaverse' before?
11. Are you familiar with what the metaverse is?
12. In the future, could patient education be provided through the Metaverse?
13. In the future, is it possible to perform virtual nursing using the Metaverse?
